# ACY1 Downregulation Enhances the Radiosensitivity of Cetuximab-Resistant Colorectal Cancer by Inactivating the Wnt/β-Catenin Signaling Pathway

**DOI:** 10.3390/cancers14225704

**Published:** 2022-11-21

**Authors:** Wulin Shan, Chunyang Dai, Huanhuan Zhang, Dan Han, Qiyi Yi, Bairong Xia

**Affiliations:** 1 Department of Laboratory Diagnostics, First Affiliated Hospital of USTC, Division of Life Sciences and Medicine, University of Science and Technology of China, Hefei 230031, China; 2 Core Unit of National Clinical Research Center for Laboratory Medicine, First Affiliated Hospital of USTC, Division of Life Sciences and Medicine, University of Science and Technology of China, Hefei 230031, China; 3 Department of Cancer Epigenetics Program, First Affiliated Hospital of USTC, Division of Life Sciences and Medicine, University of Science and Technology of China, Hefei 230031, China; 4 School of Basic Medical Sciences, Anhui Medical University, Hefei 230032, China; 5 Department of Gynecology, First Affiliated Hospital of USTC, Division of Life Sciences and Medicine, University of Science and Technology of China, Hefei 230031, China

**Keywords:** colorectal cancer, cetuximab resistance, radiosensitivity, ACY1, Wnt/β-catenin pathway

## Abstract

**Simple Summary:**

Cetuximab, which can be used in combination with other chemotherapies, is widely used as a standard of care treatment in the majority of advanced colorectal cancer (CRC) patients. However, the treatment of cetuximab-resistant CRC remains a major problem. The study was to assess the potential effects of radiotherapy on cetuximab-resistant CRC and explored the underlying mechanism. This review discussed a novel role of ACY1 in radiotherapy of cetuximab-resistant CRC, suggesting a possible target for cetuximab-resistant CRC treatment in the clinic.

**Abstract:**

Treatment of cetuximab-resistant colorectal cancer (CRC) is a global healthcare problem. This study aimed to assess the effects of radiotherapy on cetuximab-resistant CRC and explore the underlying mechanism. We established a cetuximab-resistant HCT116 cell line (HCT116-R) by extracorporeal shock. Differentially expressed mRNAs were screened from cells treated with different radiation doses using second-generation high-throughput sequencing. Sequence data showed that ACY1 was significantly downregulated in HCT116-R cells after irradiation. Analysis of the GEO and TCGA datasets revealed that high ACY1 expression was associated with lymph node metastasis and a poor prognosis in CRC patients. In addition, immunohistochemistry results from CRC patients revealed that ACY1 protein expression was related to cetuximab resistance and lymph node metastasis. These findings suggested that ACY1 may function as an oncogene to promote CRC progression and regulate the radiosensitivity of cetuximab-resistant CRC. As expected, ACY1 silencing weakened the proliferation, migration, and invasion abilities of HCT116-R cells after radiotherapy. Mechanistically, TCGA data demonstrated that ACY1 expression was closely related to the Wnt/β-catenin pathway in CRC. We validated that radiotherapy first reduced β-catenin levels, followed by decreased expression of the metastasis-related protein E-cadherin. Silencing ACY1 dramatically enhanced these changes in β-catenin and E-cadherin after radiotherapy. In conclusion, ACY1 downregulation could enhance the radiosensitivity of cetuximab-resistant CRC by inactivating Wnt/β-catenin signaling, implying that ACY1 may serve as a radiotherapy target for cetuximab-resistant CRC.

## 1. Introduction

Colorectal cancer (CRC) is one of the most commonly identified types of cancer, with an increasing incidence worldwide [1]. The high mortality of CRC is partially attributed to the advanced stage at diagnosis, leading to metastasis and recurrence over a short period [2,3]. Approximately 25% of patients already have metastasis at the time of diagnosis, and 50% will eventually develop metastatic CRC (mCRC) [4]. Advancements in the understanding of mCRC pathophysiology have led to increased treatment options, including radiotherapy, immunotherapy, palliative chemotherapy, targeted therapy, extensive surgery, and local ablative therapies for metastases [5]. Cetuximab is a recombinant, chimeric, monoclonal antibody that specifically targets the extracellular domain of human epidermal growth factor receptors and is currently the standard regimen for the first-line treatment of mCRC [6,7]. However, approximately 80% of mCRC patients who harbor KRAS, NRAS, BRAF, and PIK3CA gene mutations cannot benefit from cetuximab treatment [8]. Additionally, disease progression will occur in almost all patients who are initially sensitive to cetuximab within 3–12 months [9]. It is therefore necessary to identify other effective approaches for treating cetuximab-resistant CRC. Preoperative radiotherapy has been included in the local treatment of advanced CRC [10,11], and more effective radiosensitizing agents are currently being explored [12]. The present study aimed to explore the efficacy and underlying mechanism of radiotherapy for cetuximab-resistant CRC.

Aminoacylase 1 (ACY1) is a cytosolic enzyme that is widely distributed in mammalian tissues and catalyzes the hydrolysis of acylated amino acids into amino acids and acyl groups [13]. N-terminal acetylation is a widespread and highly conserved process involved in the protection and stability of proteins [14]. The main functions of ACY1 are to accelerate the hydrolysis of N-acetylated peptides, especially N-acetylated neutral aliphatic amino acids, and participate in protein synthesis and turnover through the release of free amino acids [15]. Several studies have shown that ACY1 plays an important role in the pathogenesis of numerous tumors, such as colorectal, liver, small cell lung, and renal cell cancers [16,17]. However, the functions of ACY1 differ among tumors. ACY1 expression was significantly reduced in small cell lung and renal cell carcinomas, suggesting that it acted to inhibit tumorigenesis [18]. Conversely, however, ACY1 was overexpressed in liver cancer, and ACY1 levels were associated with tumor proliferation, invasion, and metastasis [19]. Several studies also showed that ACY1 protein was upregulated in CRC compared with normal colon tissues [17,20]. However, the association between ACY1 expression and radiosensitivity in cetuximab-resistant CRC remains unclear.

The present study showed that radiotherapy had a significant anticancer effect on cetuximab-resistant CRC cells and that ACY1 expression was markedly decreased after radiation. Analysis of GEO and TCGA datasets showed that high ACY1 levels were related to poor prognosis and lymph node metastasis in patients with CRC. These results suggest that ACY1 may be an important oncogene regulating the radiosensitivity of cetuximab-resistant CRC. Consistent with our prediction, silencing ACY1 effectively inhibited the proliferation, migration, and invasion of cetuximab-resistant CRC cells after radiotherapy. Finally, radiotherapy dramatically inactivated the Wnt/β-catenin signaling pathway in cetuximab-resistant CRC cells. Altogether, our study highlighted a novel role of ACY1 in the radiotherapy of cetuximab-resistant CRC, suggesting a possible target for cetuximab-resistant CRC treatment in the clinic.

## 2. Methods

### 2.1. Patients and Clinical Tissue Samples

Fifteen paired CRC and adjacent normal tissue samples were obtained from patients with CRC who underwent surgery in the First Affiliated Hospital of USTC (Hefei, China) from 2018 to 2021 and who did not receive any form of chemoradiotherapy. All tissue samples were stored at −80 °C immediately after resection. The study was approved by the Medical Ethics Committee of the First Affiliated Hospital of USTC. Informed consent was obtained from all participating patients prior to initiation or enrollment in the present study.

### 2.2. Cell Lines and Cell Culture

The human CRC cell line HCT116 was purchased from the Cancer Institute of the Chinese Academy of Medical Science and grown in RPMI 1640 Medium (Thermo Fisher Scientific, Cambridge, MA, USA) supplemented with 10% fetal bovine serum (Thermo Fisher Scientific, Cambridge, MA, USA) and 1% penicillin–streptomycin (Thermo Fisher Scientific, Cambridge, MA, USA). All cells were cultured in a humidified incubator with 5% CO_2_ and 95% air at 37 °C. A cetuximab-resistant HCT116 cell line (HCT116-R) was induced by extracorporeal shock in our own laboratory. Specifically, HCT116 cells were cultured in medium at a final concentration of 20 μg/mL cetuximab for 48 h. Adherent cells were then cultured in medium without cetuximab until they gradually recovered (approximately 24 h). The cell state returned after one generation, and 20 μg/mL cetuximab was then administered again. The same procedure was repeated approximately eight times, and the HCT116-R cell line was finally established.

### 2.3. Cell Viability Assay

Cells in log growth phase were seeded in 96-well plates at a density of 8 × 10^3^/well (in triplicate) to allow adhesion. The cells were incubated with graded concentrations (0–20 μg/mL) of cetuximab and cultured for 24 and 48 h, followed by the addition of 10 µL CCK-8 reagent (Abcam, Cambridge, UK) for an extra 2 h. Cell viability was determined by measuring the optical density at 450 nm using a microplate reader (Tecan Group Ltd., Salzburg, Austria) at 450 nm. The cells were then cultured with fresh medium until the next round of measurement.

### 2.4. X-ray Irradiation

The radiotherapy equipment included an American Varian TrueBeam linear accelerator (Varian, Palo Alto, CA, USA). Cells were irradiated with various doses according to the set parameters. Cells were irradiated with graded doses of X-rays (0, 4, and 6 Gy).

### 2.5. Colony Formation Assay

Cells were seeded into 6-well plates, incubated for 12 days, washed with phosphate-buffered saline (PBS) and fixed with 4% paraformaldehyde for 30 min. Then, 0.1% crystal violet was added to the plates, which were further incubated at room temperature for 20 min. Finally, the cells were washed with PBS, dried, and imaged.

### 2.6. High-Throughput Sequencing

Total RNA was isolated from HCT116-R cells treated with 0, 4, and 6 Gy irradiation using a mirVana miRNA Isolation Kit (Applied Biosystems, Foster City, LA, USA). A total of 1.5 µg RNA per sample was used as input material for RNA sample preparations. Sequencing libraries were generated using a small RNA Sample Library Prep Kit for Illumina (New England Biolabs, Ipswich, UK) following the manufacturer’s instructions, and index codes were added to attribute sequences to each sample. Genes with *p* < 0.05 and fold change >2 were considered differentially expressed genes.

### 2.7. Bioinformatics Analysis

All CRC clinical data and copy numbers in TCGA colon adenocarcinoma (COAD) and rectum adenocarcinoma (READ) datasets were retrieved through UCSC XENA (https://xenabrowser.net, accessed on 12 November 2021) [21]. TIMER (https://cistrome.shinyapps.io/timer, accessed on 15 November 2021) was used to explore the differential expression of ACY1 between tumor and adjacent noncancerous samples across all TCGA tumors [22]. In addition, we systematically searched for publicly available CRC datasets and reported clinical annotations in GEO, and downloaded the data. The GEO datasets GSE3629, GSE37182, GSE4459, GSE38832, GSE40967, GSE46862, and GSE87211 were used in this study. The association between ACY1 expression levels and the Wnt/β-catenin pathway in CRC was explored by integrated analysis of the TCGA database.

### 2.8. Immunohistochemistry

All CRC samples were fixed in formalin, embedded in paraffin, and cut into 4 μm sections. The sections were deparaffinized in xylene followed by microwave treatment for 10 min in PBS, pH 6.0. After cooling for 20 min and washing with PBS, the sections were incubated at room temperature for 10 min to inactivate endogenous peroxidase, washed with PBS, and incubated in nonimmune animal serum for 60 min to reduce nonspecific antibody binding. The sections were then incubated overnight at 4 °C with anti-ACY1 (1:300; Santa Cruz, CA, USA). Following the manufacturer’s instructions, immunostaining was performed using an UltraSensitive™ S-P kit (Kalang, Shanghai, China) with 3,3-diamino-benzidine (Santa Cruz) as the chromogen. Finally, the slices were redyed with hematoxylin, dehydrated, cleared, and covered with glass.

### 2.9. Wound-Healing Assay

Cells were scraped off the 6-well plates using 200 μL pipette tips. The plates were then washed with PBS and incubated for 24 h, and the migration distance of the cells was observed under a phase-contrast microscope at 0 and 24 h and measured using ImageJ software (version 1.43 National Institutes of Health, Bethesda, MD, USA).

### 2.10. Cell Invasion Assay

Cells were seeded onto the upper level of a transwell chamber coated with Matrigel (BD Biosciences, San Jose, CA, USA) and cultured in serum-free RPMI 1640 medium. RPMI 1640 medium supplemented with 10% fetal bovine serum was added as a chemoattractant to the lower chamber. After 48 h, the cells attached to the lower surface of the chamber were fixed for 30 min with 20% methanol and stained for 20 min with 0.1% crystal violet. Cells in three different fields of vision were then counted, and the results were used to determine the average number of cells. All experiments were performed independently three times.

### 2.11. RNA Extraction and Real-Time PCR Assay Analysis

Total RNA was extracted using TRIzol reagent (TaKaRa, Shiga, Japan), and cDNA was synthesized using the PrimeScript RT–PCR Kit (TaKaRa, Shiga, Japan). Real-time quantitative PCR was performed using SYBR Green Master Mix (TaKaRa, Shiga, Japan) on a LightCycler 96 Detection System (Roche, Mannheim, Germany). The cDNA was used to conduct real-time PCR using the following primers: ACY1 forward: 5′-GGCTGCATGAGGCTGTGTT-3′, reverse: 5′-CTTGGCACTGGTTGGGATG-3′; and glyceraldehyde 3-phosphate dehydrogenase (GAPDH) forward: 5′-TGGCACCCAGCACAATGAA-3′, reverse: 5′-CTAAGTCATAGTCCGCCTAGAAGCA-3′. The mRNA expression of ACY1 was calculated using the 2^−ΔΔCt^ method.

### 2.12. Cell Transfection

HCT116-R cells were transfected with small interfering RNA (siRNA) (Shanghai GeneChem, Shanghai, China) targeting ACY1 using Lipofectamine™ 2000 (Invitrogen, Carlsbad, CA, USA) following the manufacturer’s recommendations. The cells were transfected for 24 h for further analysis.

### 2.13. Western Blotting

Lysates from CRC cells were separated on 10% sodium dodecyl sulfate-polyacrylamide gels, and the proteins were then transferred to a polyvinylidene difluoride membrane (Millipore, Billerica, MA, USA). The membranes were blocked with TBST (0.5% Tween-20 in Tris-buffered saline) containing 5% nonfat milk for 1 h at room temperature and then incubated with primary antibody overnight at 4 °C, followed by secondary antibodies for 1 h at room temperature. The following antibodies were used: anti-GAPDH, anti-ACY1, anti-E-cadherin, and β-catenin (Cell Signaling Technology, Boston, MA, USA). The samples were visualized using an enhanced chemiluminescence system (Thermo Fisher Scientific, Cambridge, MA, USA), and the bands were analyzed with ImageJ software (version 1.43 National Institutes of Health, Bethesda, MD, USA).

### 2.14. Statistical Analyses

Data were presented as the mean and standard deviation. All statistical analyses were performed using GraphPad Prism (GraphPad Software Inc., La Jolla, CA, USA). The significance of the results was calculated by two-way ANOVA or two-tailed Student’s *t* test. A *p* value < 0.05 was considered to be significant.

## 3. Results

### 3.1. Irradiation Treatment Significantly Reduced ACY1 Expression in Cetuximab-Resistant CRC Cells

**Figure 1:**
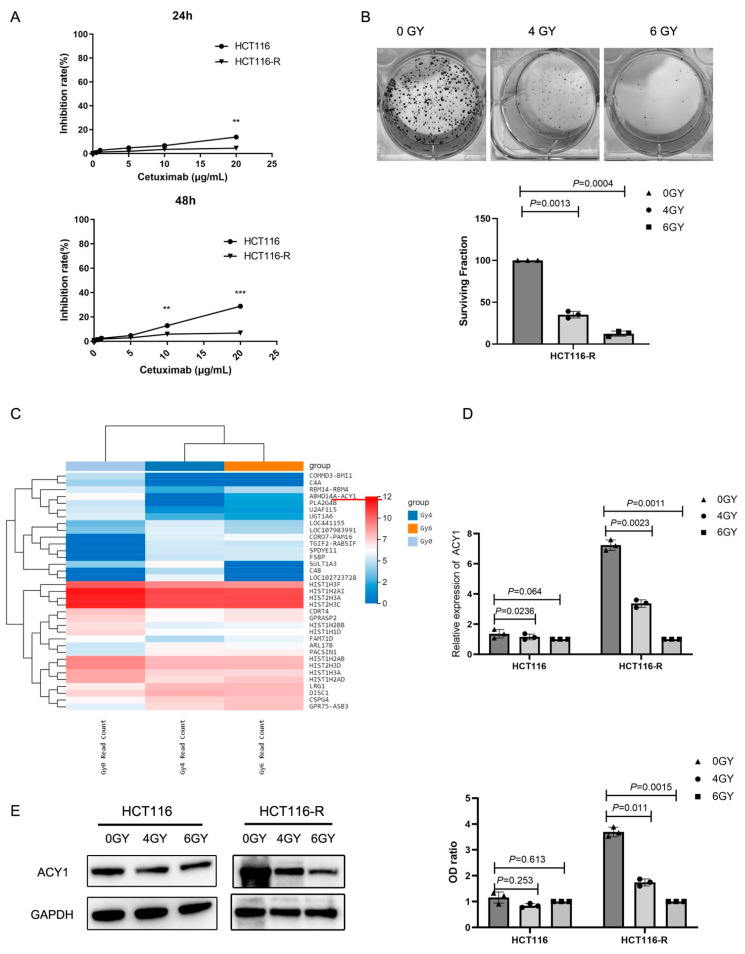
Irradiation treatment significantly inhibits the proliferation and reduces the expression of ACY1 in cetuximab-resistant CRC cells. (**A**) The sensitivity of a panel of the CRC cell line HCT116 and the cetuximab-resistant CRC cell line HCT116-R to cetuximab was assessed by CCK8 assays. CRC cells were incubated with graded concentrations (0–20 μg/mL) of cetuximab for 24 h and 48 h. (**B**) A colony formation assay was performed in HCT116-R cells after exposure to graded doses of irradiation (0, 4, and 6 Gy). The surviving fraction of HCT116-R cells was determined at least three times, and the data are presented as the mean ± SD. (**C**) The heatmap from the hierarchical clustering of 35 mRNA expression patterns in HCT116-R cells treated with 0, 4, and 6 Gy irradiation is shown. (**D**) The expression of ACY1 was examined by qRT-PCR in HCT116 and HCT116-R cells treated with 0, 4, and 6 Gy irradiation. The experiment was repeated at least three times, and the data are presented as the mean ± SD. (**E**) The expression of ACY1 in HCT116 and HCT116-R cells treated with 0, 4, and 6 Gy irradiation was examined by Western blotting. The experiment was repeated at least three times, and the data are presented as the mean ± SD. ** *p* < 0.01, and *** *p* < 0.001.

We first established a panel of cetuximab-resistant CRC cells from HCT116 cells by extracorporeal shock. The cetuximab-resistant subpopulation was designated HCT116-R. Compared with HCT116 cells, HCT116-R cells showed greater resistance to cetuximab (Figure 1A). To explore the efficacy of radiotherapy in cetuximab-resistant CRC, we irradiated HCT116-R cells with 4 and 6 Gy and observed the effects on cell proliferation. A colony formation assay, a standard method for detecting radiosensitivity [23], showed that the surviving fraction of HCT116-R cells decreased significantly with increasing radiation dose (Figure 1B). Compared with unirradiated HCT116-R cells, the surviving fractions of HCT116-R cells after irradiation with 4 and 6 Gy were 15.0 ± 1.62 and 3.67 ± 0.94, respectively, indicating that radiotherapy was effective in cetuximab-resistant CRC.

We further explored the regulatory molecules during radiotherapy by high-throughput sequencing in HCT116-R cells treated with 0, 4, and 6 Gy irradiation. The heatmap of hierarchical clustering of mRNA expression profiles is shown in Figure 1C. A total of 21 mRNAs (*p* < 0.05, fold-change > 2.0) were upregulated, and 18 mRNAs (*p* < 0.05, fold change > 2.0) were downregulated compared with the unirradiated HCT116-R cells. Notably, ACY1 was estimated to be the most highly downregulated gene in HCT116-R cells after irradiation. We confirmed these results by real-time PCR (Figure 1D) and Western blotting (Figure 1E), which showed similar trends. However, the expression level of ACY1 in HCT116 cells did not decrease with the increase in radiation dose (Figure 1D,E). These results suggested that ACY1 might be a key factor regulating the radiosensitivity of cetuximab-resistant CRC.

### 3.2. ACY1 Levels Were Associated with Tumor Metastasis, Prognosis, and Cetuximab Resistance in CRC

**Figure 2:**
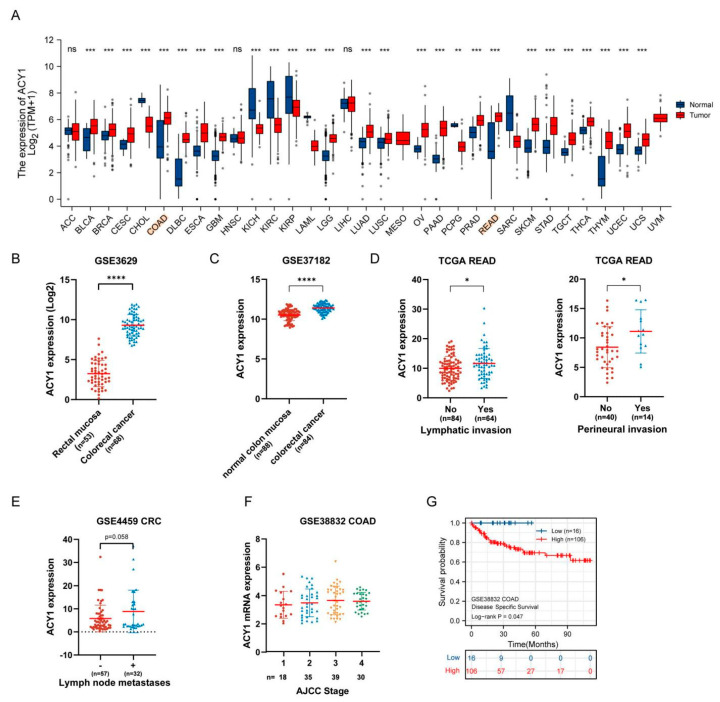
ACY1 expression in human cancers and its relationship with clinical severity stratification and prognosis in CRC patients. (**A**) ACY1 expression levels in diverse tumors and adjacent noncancerous tissues from TCGA database. Comparison of ACY1 expression in tumors and corresponding normal tissues in different types of cancer. (Red indicates tumor samples; blue indicates noncancerous samples). ACC, adrenocortical carcinoma; BLCA, bladder urothelial carcinoma; BRCA, breast invasive carcinoma; CESC, cervical squamous cell carcinoma and endocervical adenocarcinoma; CHOL, cholangiocarcinoma; COAD, colon adenocarcinoma; DLBC, lymphoid neoplasm diffuse large B-cell lymphoma; ESCA, esophageal carcinoma; GBM, glioblastoma multiforme; HNSC, head and neck squamous cell carcinoma; KICH, kidney chromophobe; KIRC, kidney renal clear cell carcinoma; KIRP, kidney renal papillary cell carcinoma; LAML, acute myeloid leukemia; LGG, brain lower grade glioma; LIHC, liver hepatocellular carcinoma; LUAD, lung adenocarcinoma; LUSC, lung squamous cell carcinoma; MESO, mesothelioma; OV, ovarian serous cystadenocarcinoma; PAAD, pancreatic adenocarcinoma; PCPG, pheochromocytoma and paraganglioma; PRAD, prostate adenocarcinoma; READ, rectum adenocarcinoma; SARC, sarcoma; SKCM, skin cutaneous melanoma; STAD, stomach adenocarcinoma; TGCT, testicular germ cell tumors; THCA, thyroid carcinoma; THYM, thymoma; UCEC, uterine corpus endometrial carcinoma; UCS, uterine carcinosarcoma; UVM, uveal melanoma. (**B**) human ACY1 expression in normal rectal mucosa and colorectal cancer (including low-grade dysplasia and high-grade dysplasia) from GSE3629 datasets. (**C**) human ACY1 expression in normal colon mucosa and colorectal cancer (including low-grade dysplasia and high-grade dysplasia) from GSE37182 datasets. (**D**) ACY1 expression levels in READ patients with lymphatic invasion or not and with perineural invasion or not from TCGA datasets. (**E**) ACY1 expression levels in CRC patients with or without lymph node metastases from the GSE4459 dataset. (**F**) ACY1 expression levels in COAD patients with different AJCC stages in the GSE38832 dataset. (**G**) Kaplan–Meier analysis of DSS in GSE38832 COAD patients based on ACY1 expression. DSS, disease-specific survival; * *p* < 0.05, ** *p* < 0.01, *** *p* < 0.001 and **** *p* < 0.0001. ns. means no significance.

We explored the potential role of ACY1 in CRC progression by assessing its expression profiles in tumor and adjacent noncancerous tissues in TCGA datasets using the “DiffExp module” of TIMER. Among 33 tumors, ACY1 expression was markedly higher in 22 types of tumors compared with adjacent noncancerous samples, including adrenocortical carcinoma, bladder urothelial carcinoma, breast invasive carcinoma, cervical squamous cell carcinoma and endocervical adenocarcinoma, lymphoid neoplasm diffuse large B-cell lymphoma, lung adenocarcinoma, and lung squamous cell carcinoma (Figure 2A). Similarly, ACY1 levels were significantly higher in colon adenocarcinoma (COAD) and rectum adenocarcinoma (READ) tissues than in adjacent noncancerous samples (Figure 2A), suggesting a general tumor-activator role of ACY1. Moreover, higher expression of ACY1 in CRC was confirmed in the GEO GSE3629 and GSE37182 datasets (Figure 2B,C).

We further explored the impact of ACY1 on CRC progression by analyzing the correlation between ACY1 expression and clinical parameters in CRC patients in TCGA and GEO datasets, including COAD and READ. High ACY1 expression was associated with lymphatic invasion and perineural invasion in READ in TCGA datasets (Figure 2D), lymph node metastases in CRC in the GSE4459 dataset (Figure 2E), and the American Joint Committee on Cancer stage (AJCC) for COAD in the GSE38832 dataset (Figure 2F). However, there were no obvious correlations between ACY1 expression and pathologic TNM stage, the presence of colon polyps, or residual tumor for CRC in TCGA datasets (Figures S1A–D and S2A–D).

The prognostic value of ACY1 expression in CRC was also evaluated by Kaplan–Meier analysis, which showed that high ACY1 expression was correlated with shorter DSS (*p* = 0.047) in GSE38832 COAD (Figure 2G), with shorter progression-free interval (PFI) (*p* = 0.072) and disease-specific survival (DSS) (*p* = 0.263) in TCGA COAD, and with shorter recurrence-free survival (RFS) (*p* = 0.091) in GSE40967 COAD (Figure S1E). Although some of the Kaplan–Meier results were not significant, there was a corresponding trend. These data thus indicated a poor prognosis in patients with high ACY1 expression.

**Figure 3:**
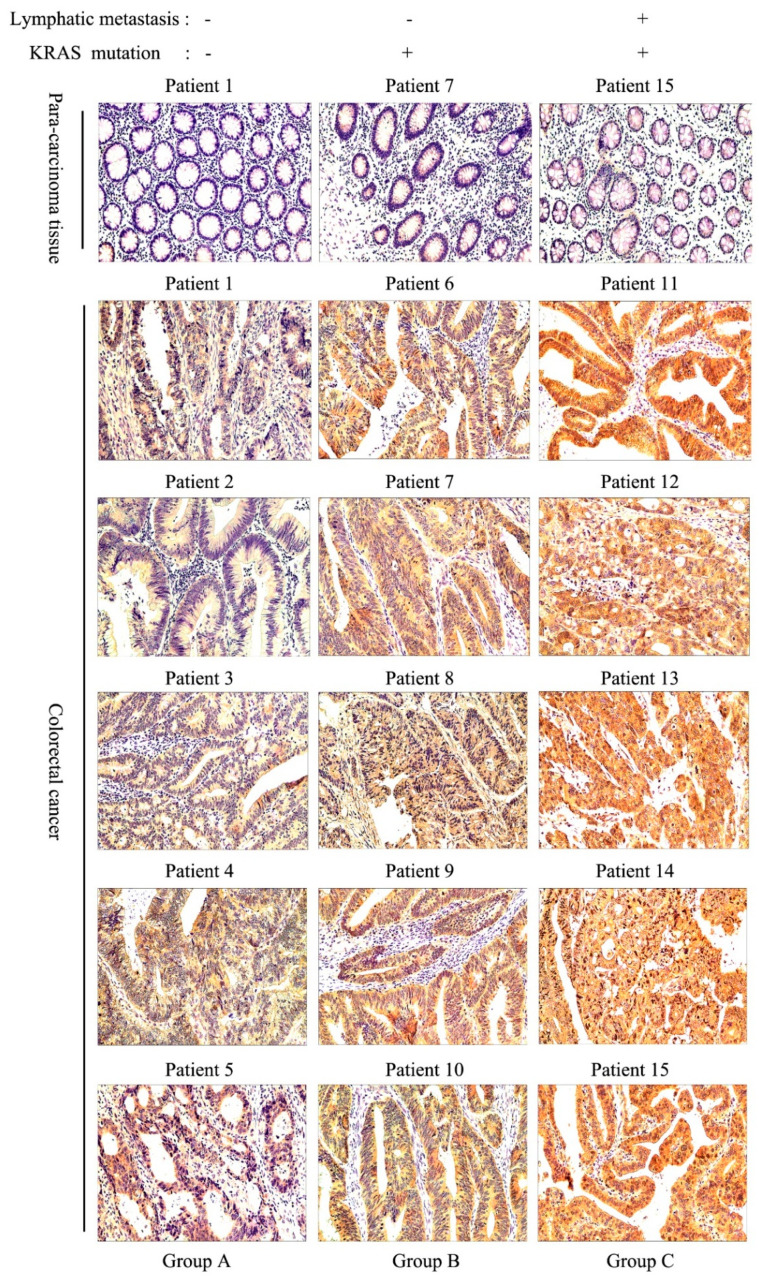
High expression of ACY1 is associated with lymph node metastasis and cetuximab resistance in CRC. Representative IHC images of the expression of ACY1 in tissue samples from CRC patients. Patient #1~#5: lymph node metastasis negative, KRAS wild type. Patient #6~#10: lymph node metastasis positive, KRAS wild type. Patient #11~#15: lymph node metastasis positive, KRAS mutant type. KRAS mutation indicates cetuximab resistance in CRC.

To verify whether ACY1 expression was related to lymph node metastasis and cetuximab resistance in CRC, we selected 15 CRC patients and divided them into three groups: no lymph node metastasis, *KRAS* wild-type (Patients #1–#5, Group A); no lymph node metastasis, *KRAS* mutant-type (Patients #6–#10, Group B); and lymph node metastasis, *KRAS* mutant-type (Patients #11–#15, Group C). The clinical information for these 15 patients is shown in Table 1. H&E staining was performed to observe the tumor tissue of all 15 CRC patients and lymph node metastasis of Patients #11–#15 (Figure S3). *KRAS* mutation has been shown to be a predictor of resistance to cetuximab therapy [24,25], and *KRAS* mutation thus implied cetuximab resistance in our study. ACY1 expression levels were negative or low in paracancerous tissues in each group and highest in CRC patients with lymph node metastases and *KRAS* mutation (group C) (Figure 3). In addition, ACY1 expression levels were lower in patients with *KRAS* mutation but no lymph node metastasis (group B) than in group C. The expression levels were lowest in patients without lymph node metastasis and *KRAS* mutation (group A). These results suggested that ACY1 was involved in the processes of lymph node metastasis and cetuximab resistance in CRC patients.

### 3.3. ACY1 Was Involved in Regulating the Radiosensitivity of Cetuximab-Resistant CRC

**Figure 4:**
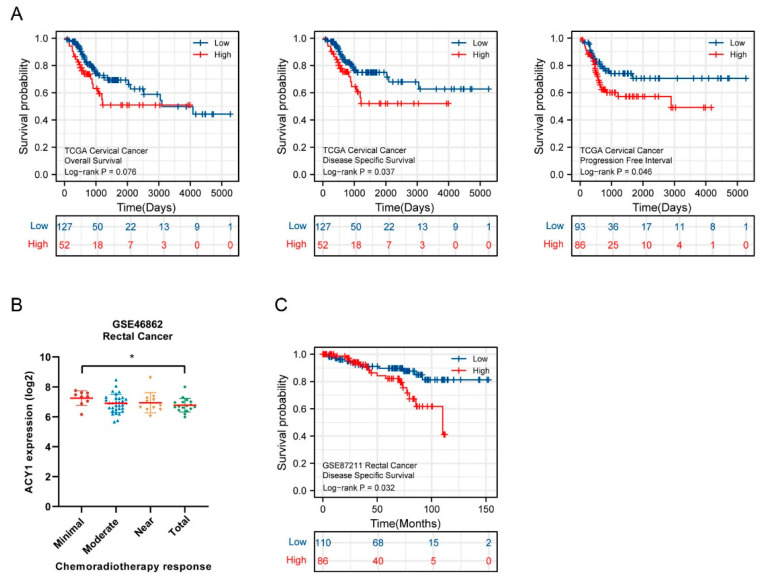
High ACY1 expression predicts poor efficacy of radiotherapy and worse prognosis in patients with cervical or rectal cancer. (**A**) Kaplan–Meier analysis of OS, DSS and PFI in cervical cancer patients with radiotherapy based on ACY1 expression from TCGA datasets. (**B**) The relationship between the expression of ACY1 and the response to chemoradiotherapy in rectal cancer patients from the GSE46862 dataset. (**C**) Kaplan–Meier analysis of DSS in rectal cancer patients based on ACY1 expression from the GSE87211 dataset. OS, overall survival; DSS, disease-specific survival; PFI, progression-free interval. * *p* < 0.05.

We further explored the impact of ACY1 on tumor radiotherapy by analyzing related research in TCGA and GEO datasets. Data on cervical cancer in TCGA showed worse overall survival (*p* = 0.076), DSS (*p* = 0.037), and PFI following radiotherapy (*p* = 0.046) in patients with high ACY1 expression (Figure 4A). We also examined the correlation between baseline ACY1 expression levels and tumor response to chemoradiotherapy in patients with rectal cancer. The response to chemoradiotherapy can be classified as minimal, moderate, near, or total. ACY1 expression decreased with increasing chemoradiotherapy response in patients with rectal cancer in the GSE46862 dataset (Figure 4B). We also evaluated the association between the prognosis of radiotherapy and ACY1 expression in patients with rectal cancer and showed that high ACY1 expression was significantly correlated with shorter DSS (*p* = 0.032) (Figure 4C). Overall, these results suggested that ACY1 might be involved in the response to radiotherapy in cervical and rectal cancers. However, there was no information on the correlation between radiotherapy and ACY1 expression levels in patients with colon cancer in TCGA and GEO datasets.

**Figure 5:**
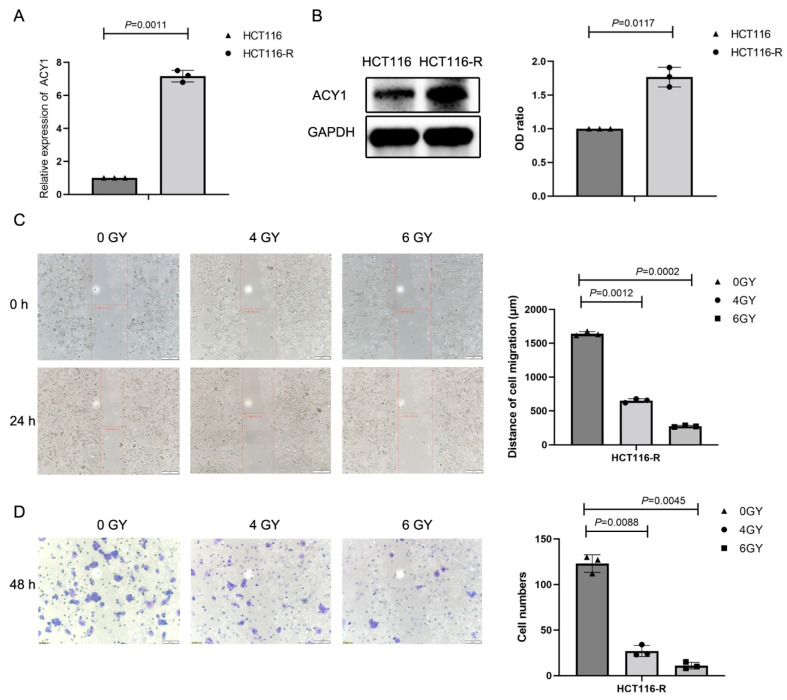
Radiotherapy affects the migration and invasion ability of HCT116-R cells. (**A**) The expression of ACY1 in HCT116 and HCT116-R cells was examined by qRT–PCR. (**B**) The expression of ACY1 in HCT116 and HCT116-R cells was examined by Western blotting. (**C**) Wound healing migration assay showing the impact of different doses of radiotherapy on HCT116-R cells (24 h). (**D**) Invasion assay showing the impact of different doses of radiotherapy on HCT116-R cells (48 h). All experiments were repeated at least three times, and the data are presented as the mean ± SD.

We showed that ACY1 expression decreased with increasing radiation dose in HCT116-R cells (Figure 1D,E). Combining the results of Figure 1 and Figure 4, we speculated that ACY1 might affect the radiosensitivity of cetuximab-resistant CRC. To confirm this hypothesis, we explored ACY1 expression in HCT116 and HCT116-R CRC cells by real-time PCR (Figure 5A) and Western blotting (Figure 5B). ACY1 expression was significantly upregulated in HCT116-R cells compared with HCT116 cells, in accord with the results that ACY1 is associated with cetuximab resistance in HCT116 cells, as shown in Figure 3.

Given that ACY1 was mainly associated with lymph node metastasis and perineural invasion in CRC (Figure 2D,E), we investigated the effects of radiotherapy on the migration and invasion of HCT116-R cells. HCT116-R cells treated with 4 and 6 Gy irradiation showed reduced migration abilities compared with unirradiated cells according to wound-healing assays (Figure 5C), as well as decreased invasion ability (Figure 5D).

**Figure 6:**
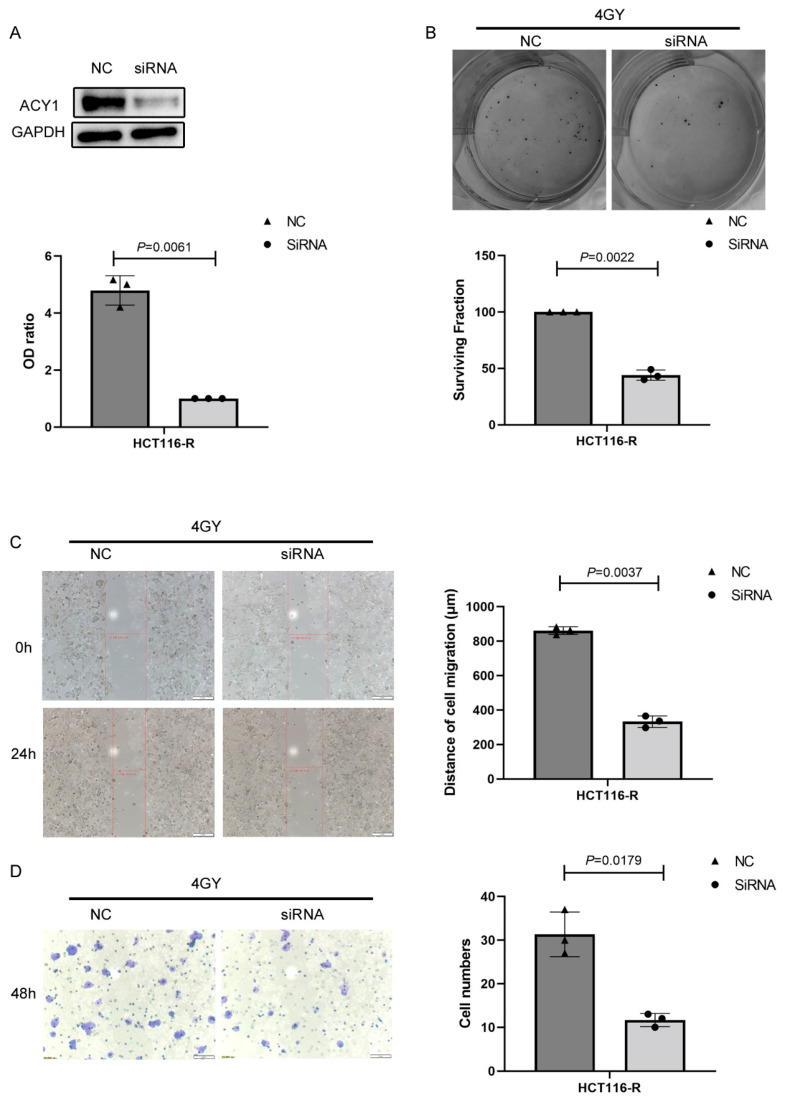
Silencing ACY1 in HCT116-R cells increased the sensitivity to radiation. (**A**) HCT116-R cells were transfected with siRNA-NC or siRNA-ACY1, and the protein levels of ACY1 were estimated by Western blotting. (**B**) Representative images and the survival fraction of colony formation in siRNA-NC or siRNA-ACY1 HCT116-R cells treated with 4 Gy irradiation. (**C**) Wound healing assay measuring the migration ability of HCT116-R cells transfected with siRNA-NC or siRNA-ACY1 at 24 h treated with 4 Gy irradiation. (**D**) Transwell invasion assays were performed in HCT116-R cells transfected with siRNA-NC or siRNA-ACY1 at 48 h and treated with 4 Gy irradiation. All experiments were repeated at least three times, and the data are presented as the mean ± SD.

To determine whether ACY1 regulated the response to radiotherapy in cetuximab-resistant CRC, we transfected HCT116-R cells with siRNA-ACY1 and control siRNA (siRNA-NC). Western blotting confirmed that intracellular ACY1 levels were markedly decreased in siRNA-ACY1-transfected HCT116-R cells compared with siRNA-NC-transfected HCT116-R cells (Figure 6A), indicating successful silencing of ACY1 by siRNA transfection. We then irradiated siRNA-NC-transfected and siRNA-ACY1-transfected HCT116-R cells with 4 Gy and showed that the surviving fraction of siRNA-ACY1-transfected HCT116-R cells was significantly lower than that of siRNA-NC-transfected cells (Figure 6B). In addition, we explored the effects of ACY1 silencing on the radiosensitivity of HCT116-R cells by wound healing and invasion assays. Compared with siRNA-NC-transfected HCT116-R cells, siRNA-ACY1-transfected cells displayed decreased migration and invasion abilities after exposure to 4 Gy radiation (Figure 6C,D). Collectively, these findings suggested that ACY1 silencing enhanced the effects of radiotherapy on cetuximab-resistant CRC.

### 3.4. ACY1 Inactivated Wnt/β-Catenin Signaling to Regulate the Radiosensitivity of Cetuximab-Resistant CRC

**Figure 7:**
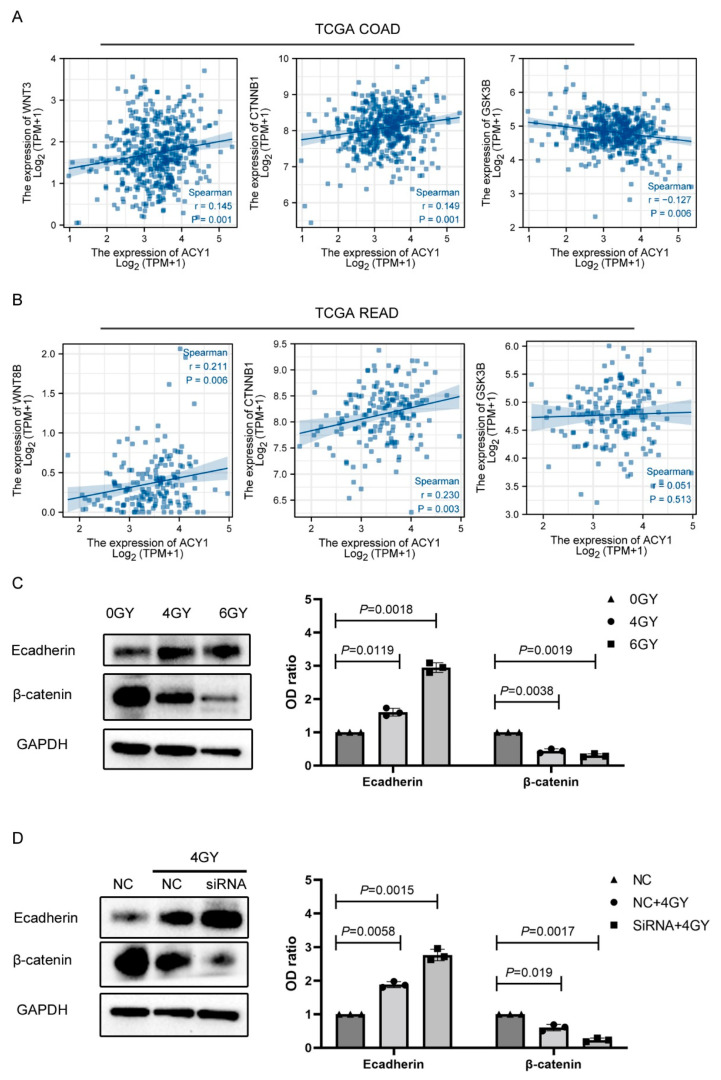
ACY1 inactivated the Wnt/β-catenin pathway to regulate the radiosensitivity of HCT116-R cells. (**A**) Spearman’s correlation analysis of ACY1 expression and WNT3, CTNNB1, and GSK3B in the TCGA COAD dataset. (**B**) Spearman’s correlation analysis of ACY1 expression and WNT8B, CTNNB1, and GSK3B in the TCGA READ dataset. (**C**) The Western blot assay revealed the expression of E-cadherin and β-catenin in HCT116-R cells treated with 0, 4, and 6 Gy. (**D**) The Western blot assay revealed that the levels of E-cadherin and β-catenin in HCT116-R cells transfected with siRNA-NC or siRNA-ACY1 treated with 4 Gy. The experiment was repeated three times, and the data are presented as the mean ± SD.

Aberrant Wnt/β-catenin signaling is central to carcinogenesis and metastasis in most tumors, particularly CRC [26,27]. We, therefore, investigated the potential involvement of the Wnt/β-catenin signaling pathway in the process of ACY1-regulated radiosensitivity in cetuximab-resistant CRC. We evaluated the correlation between ACY1 expression levels and the expression of the key Wnt/β-catenin pathway genes, including WNT3, CTNNB1, GSK3, and WNT8B, by Spearman’s correlation coefficient from TCGA COAD and READ databases. ACY1 expression was positively correlated with WNT3 (Spearman’s ρ = 0.145, *p* = 0.001) and CTNNB1 (Spearman’s ρ = 0.149, *p* = 0.001) in TCGA COAD and negatively correlated with GSK3B (Spearman’s ρ = −0.127, *p* = 0.006) in TCGA COAD (Figure 7A). In addition, ACY1 expression was positively correlated with WNT8B (Spearman’s ρ = 0.211, *p* = 0.006) and CTNNB1 (Spearman’s ρ = 0.230, *p* = 0.003) in TCGA READ (Figure 7B). These results demonstrated that ACY1 expression was closely related to the Wnt/β-catenin signaling pathway in CRC.

β-catenin is the key protein in the Wnt/β-catenin pathway, and its downstream protein, E-cadherin, is a marker of tumor migration and invasion. Loss of E-cadherin expression promotes cancer cell migration. We therefore investigated the role of the Wnt/β-catenin signaling pathway in the process of ACY1-regulated radiosensitivity in HCT116-R cells by detecting the expression of β-catenin and E-cadherin in HCT116-R cells after irradiation. E-cadherin levels in HCT116-R cells were significantly increased, while β-catenin levels were significantly decreased with increasing radiation dose (Figure 7C). We also explored the effect of ACY1 silencing on E-cadherin and β-catenin expression during radiotherapy. Compared with siRNA-NC-transfected HCT116-R cells, E-cadherin levels increased, and β-catenin levels decreased in siRNA-ACY1-transfected HCT116-R cells after irradiation (Figure 7D). These results indicated that ACY1 silencing increased the radiosensitivity of cetuximab-resistant CRC cells by attenuating the Wnt/β-catenin signaling pathway.

## 4. Discussion

CRC is a multistep disease involving the accumulation of genetic and epigenetic alterations. It is one of the most common malignancies worldwide and is frequently fatal [28,29]. The incidence of CRC is higher in highly developed countries but is currently increasing in middle- and low-income countries, associated with obesity and a shift toward a more Westernized lifestyle and diet [30,31]. Although significant progress has been made in the treatment of CRC in recent decades, its prognosis remains poor, especially in relation to distant metastasis in patients with advanced CRC. Cetuximab has been approved by the United States Food and Drug Administration and is widely used as a standard-of-care treatment in most patients with advanced CRC [32,33]. However, primary and acquired resistance severely restricts its wide application [34], and the treatment of cetuximab-resistant CRC remains a global healthcare problem. Radiotherapy is safe and effective and constitutes the cornerstone of clinical treatment for CRC patients [35]; however, the response to radiotherapy varies greatly from a complete response to complete resistance [36,37]. It is therefore crucial to conduct comprehensive studies to identify the key molecules involved in regulating the radiosensitivity of CRC. Tian et al. reported that silencing PFK1 inhibited cell proliferation and migration and enhanced radiosensitivity in CRC [38]. However, although numerous studies have been devoted to discovering predictive markers of radiosensitivity in CRC [39,40], few studies have examined the effects and underlying mechanisms of radiotherapy in cetuximab-resistant CRC.

In the present research, we established a cetuximab-resistant human CRC cell line (HCT116-R). The key strength of this established cell model was that it originated from the same source HCT116 cells, thus avoiding potential confounding factors such as differences in genetic background and inherent variations in radiosensitivity. The current results demonstrated that radiotherapy was effective against cetuximab-resistant CRC. We then explored the underlying mechanism and found that the expression levels of ACY1 were significantly reduced in HCT116-R cells treated with different doses of radiation. Despite the well-characterized aminoacylase activity of ACY1, experimental evidence regarding its role in tumor biology is controversial. ACY1 expression was shown to be decreased in liver cancer and renal cell carcinoma [41,42], but Yu et al. [43] found that ACY1 expression was positively associated with TNM stage in CRC. The current results from TCGA and GEO databases verified that high ACY1 expression levels were associated with lymph node metastasis and shorter cancer-specific survival in CRC. In addition, other studies revealed that ACY1 was upregulated in CRC patients compared with normal colon tissues, suggesting that ACY1 may play a pivotal role in the development of CRC [17,20]. Our immunohistochemical results from 15 CRC patients accordingly showed that ACY1 protein levels were markedly increased in malignant epithelial cells but not in paracancerous tissues and were related to lymph node metastasis and cetuximab resistance, suggesting a distinct function of ACY1 in malignant transformation.

To explore the impact of ACY1 on tumor radiotherapy, we analyzed related research in TCGA and GEO datasets. The data showed that high levels of ACY1 were related to poorer radiotherapy response and poor prognoses in cervical and rectal cancers. Unfortunately, no data on the correlation between radiotherapy and ACY1 expression in colon cancer patients were available in TCGA and GEO datasets. We then investigated the role of ACY1 in regulating the response to radiotherapy in cetuximab-resistant CRC. Our results first revealed that radiotherapy reduced the migration and invasion abilities of cetuximab-resistant CRC cells. Moreover, ACY1 silencing enhanced the effects of radiotherapy on cetuximab-resistant CRC. These data indicated that ACY1 was a key molecule involved in regulating the radiosensitivity of cetuximab-resistant CRC and might thus be a new target for tumor radiotherapy. Finally, we further explored the molecular mechanisms through which ACY1 influenced the effects of radiotherapy on cetuximab-resistant CRC. Several studies have shown the roles of ACY1 in diverse cancers. For instance, Chen et al. demonstrated that ACY1 regulated the proliferation, migration, and invasion of human neuroblastoma cells via the extracellular signal-regulated protein kinase 1/2 and transforming growth factor-β1 signaling pathways [44], and Xu et al. [45] revealed that targeting the ACY1 gene might regulate HER2 and TRAIL expression levels and reduce the occurrence and inhibit the development of rectal cancer. In addition, ACY1 promoted the progression of non-small cell lung cancer by activating phosphoinositide 3-kinase/Akt signaling in a phosphatase and tensin homolog-dependent manner [46]. However, few studies have focused on the mechanism of ACY1 in CRC radiotherapy. The Wnt signaling pathway was the first of several cellular signaling pathways related to CRC to be identified. Any abnormalities in the key components of this pathway may cause Wnt/β-Catenin activation, leading to increased cell proliferation and in turn to tumorigenesis [47]. Wnt signaling has also been associated with the effects of radiotherapy on tumors [48,49]. Given the tight association among Wnt signaling, radiotherapy, and CRC, we speculated that the Wnt pathway was likely to be involved in the response to radiotherapy in cetuximab-resistant CRC. We accordingly found that some key molecules in the Wnt signaling pathway were correlated with the expression of ACY1 in CRC using the TCGA database. For example, GSK-3β is an important component of the Wnt signaling pathway and participates in the phosphorylation of β-catenin. Phosphorylation of β-catenin is blocked when the Wnt/β-catenin signaling pathway is activated, and β-catenin translocates to and aggregates in the nucleus, finally activating the expression of downstream target genes [50]. The β-catenin gene CTNNB1 is a key coactivator for transcription factors of the T-cell factor/lymphoid enhancer factor family, leading to transcriptional activation of Wnt/β-catenin signaling in the nucleus and the encoded β-catenin protein [51]. We therefore considered that the molecular mechanism of ACY1-regulated cetuximab resistance in CRC was related to the Wnt signaling pathway. As expected, our results revealed that the expression of β-catenin, a key protein in the Wnt/β-catenin pathway, was significantly decreased with increasing radiation dose, while the levels of E-cadherin, a downstream protein in the Wnt/β-catenin pathway and a marker of tumor migration [52,53], were significantly increased. Finally, we found that the levels of E-cadherin were further increased, and the levels of β-catenin were further decreased in ACY1-downregulated HCT116-R cells after irradiation. These data indicated that ACY1 silencing enhanced the radiosensitivity of cetuximab-resistant CRC cells by inactivating Wnt/β-catenin signaling.

This study had some limitations. First, our results were based on CRC cells, which may not completely reflect physiological events in vivo. Second, the number of patients in the immunohistochemistry study was relatively small, and further clinical studies with large sample sizes should be conducted in the future. Third, this study clarified the effect and mechanism of radiotherapy on cetuximab-resistant CRC cells. However, the difference between the effects of radiotherapy on HCT116 and HCT116-R has not been clarified. This point needs to be concerned and elaborated on in the future, so as to provide a more substantial theoretical basis for the treatment of cetuximab-resistant CRC. Additionally, Pouya et al. [54] demonstrated that the effect of combining radiotherapy and cetuximab was better in CRC. Whether the combined therapy has a synergistic effect in cetuximab-resistant CRC remains unclear and could be verified in the future. Notwithstanding these limitations, this was the first study to demonstrate a correlation between the expression level of ACY1 and radiosensitivity in cetuximab-resistant CRC.

**Figure 8:**
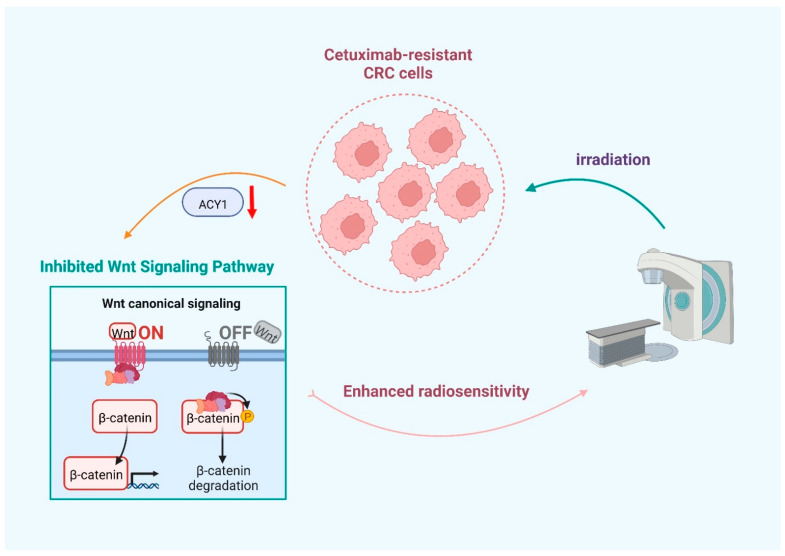
Schematic of ACY1 downregulation enhances the radiosensitivity of cetuximab-resistant colorectal cancer by inactivating the Wnt/β-catenin signaling pathway.

In summary, we identified a novel role for ACY1 as an oncogene to regulate the radiosensitivity of cetuximab-resistant CRC via the Wnt/β-catenin signaling pathway (Figure 8). These findings indicate that ACY1 might be an important target for radiotherapy in patients with cetuximab-resistant CRC.

## 5. Conclusions

The present study demonstrated a correlation between the expression level of ACY1 and radiosensitivity in cetuximab-resistant CRC, indicating that ACY1 may be a promising therapeutic agent for cetuximab-resistant CRC. In addition, we found that the Wnt/β-catenin signaling pathway is involved in regulating the ACY1 efficacy. In this work, we provided a good direction for exploring radiosensitizers in cetuximab-resistant CRC patients.

## Figures and Tables

**Table 1:** Clinical information and pathological stage of 15 patients with CRC.

Group	No. of Patient	Sex	Age	Lymphatic Metastasis	KRAS Mutation	TNM Stage	Pathological Grades
A	P1	M	63	N	Wild type	II	Middle
	P2	F	82	N	Wild type	II	Middle
	P3	M	55	N	Wild type	II	Middle-Low
	P4	M	67	N	Wild type	II	Middle
	P5	F	43	N	Wild type	I	Middle
B	P6	M	67	N	Mutant type	II	Middle
	P7	M	48	N	Mutant type	III	Middle-Low
	P8	F	49	N	Mutant type	II	Middle-Low
	P9	M	67	N	Mutant type	IV	Middle
	P10	M	75	N	Mutant type	I	Middle
C	P11	M	49	P	Mutant type	III	Middle
	P12	M	55	P	Mutant type	IV	Middle
	P13	M	69	P	Mutant type	IV	Low
	P14	M	61	P	Mutant type	IV	Low
	P15	F	57	P	Mutant type	III	Middle-Low

M: male; F: female; N: negative; P: positive; Group A: P1–P5; Group B: P6–P10; Group C: P11–P15.

## Data Availability

The data analyzed in this study were obtained from Gene Expression Omnibus (GEO) and The Cancer Genome Atlas (TCGA). The human sequence data generated in this study were not publicly available due to patient privacy requirements but were available upon reasonable request from the corresponding author. Other data generated in this study are available within the article and its supplementary data files.

## Supplementary Materials

The following supporting information can be downloaded at: https://www.mdpi.com/article/10.3390/cancers14225704/s1, Figure S1: (**A**) Relationship between the expression level of ACY1 and pathological TNM stage in TCGA COAD datasets. (**B**) Relationship between the expression level of ACY1 and pathological stage in TCGA COAD datasets. (**C**) Relationship between the expression level of ACY1 and colon polyps present in TCGA COAD datasets. (**D**) Relationship between the expression level of ACY1 and residual tumor in TCGA COAD datasets (R0 resection refers to the complete removal of the tumor in the operation, and the cutting edge is also negative when observed under the microscope; R1 resection refers to the removal of the tumor with the naked eye during the operation, but when observed under the microscope, the residual tumor cells can also be seen at the cutting edge; R2 resection means that the tumor can be seen by the naked eye during the operation, but it is not completely removed). E. Kaplan–Meier analysis of DSS and PFI in TCGA COAD patients and RFS in GSE40967 COAD patients based on ACY1 expression. DSS, disease-specific survival; PFI, progression-free interval; RFS, recurrence-free survival; Figure S2: (**A**) Relationship between the expression level of ACY1 and pathological TNM stage in TCGA READ datasets. (**B**) Relationship between the expression level of ACY1 and pathological stage in TCGA READ datasets. (**C**) Relationship between the expression level of ACY1 and colon polyps present in TCGA READ datasets. (**D**) Relationship between the expression level of ACY1 and residual tumor in TCGA READ datasets (R0 resection refers to the complete removal of the tumor in the operation, and the cutting edge is also negative when observed under the microscope; R1 resection refers to the removal of the tumor with the naked eye during the operation, but when observed under the microscope, the residual tumor cells can also be seen at the cutting edge; R2 resection means that the tumor can be seen by the naked eye during the operation, but it is not completely removed); Figure S3: H&E staining was performed to observe the tumor tissue of all 15 CRC patients and lymph node metastasis of Patients #11–#15.

## Abbreviations

ACY1: aminoacylase 1; CRC: colorectal cancer; TCGA: The Cancer Genome Atlas; GEO: Gene Expression Omnibus; mCRC: metastatic colorectal cancer; USTC: University of Science and Technology of China; Gy: Gray; RPMI: Roswell Park Memorial Institute; PBS: phosphate-buffered saline; RT–PCR: reverse transcription polymerase chain reaction; TBST: Tris-buffered saline and Tween 20; COAD: colon adenocarcinoma; READ: rectum adenocarcinoma; RNA: ribonucleic acid; siRNA: small interfering RNA; DSS: disease-specific survival; PFI: progression-free interval; NC: normal control; TNM: tumor node metastasis; GAPDH: glyceraldehyde-3-phosphate dehydrogenase; PIK3CA: phosphatidylinositol-4,5-bisphosphate 3-kinase, catalytic subunit alpha; PFK1: phosphofructokinase-1; GSK-3β: glycogen synthase kinase-3β.
