# Supplementary figures and images for: ACY1 Downregulation Enhances the Radiosensitivity of Cetuximab-Resistant Colorectal Cancer by Inactivating the Wnt/β-Catenin Signaling Pathway
Source: Cancers (Basel). 2022 Nov 21;14(22):5704. doi: 10.3390/cancers14225704 (PMC9688869; doi:10.3390/cancers14225704)

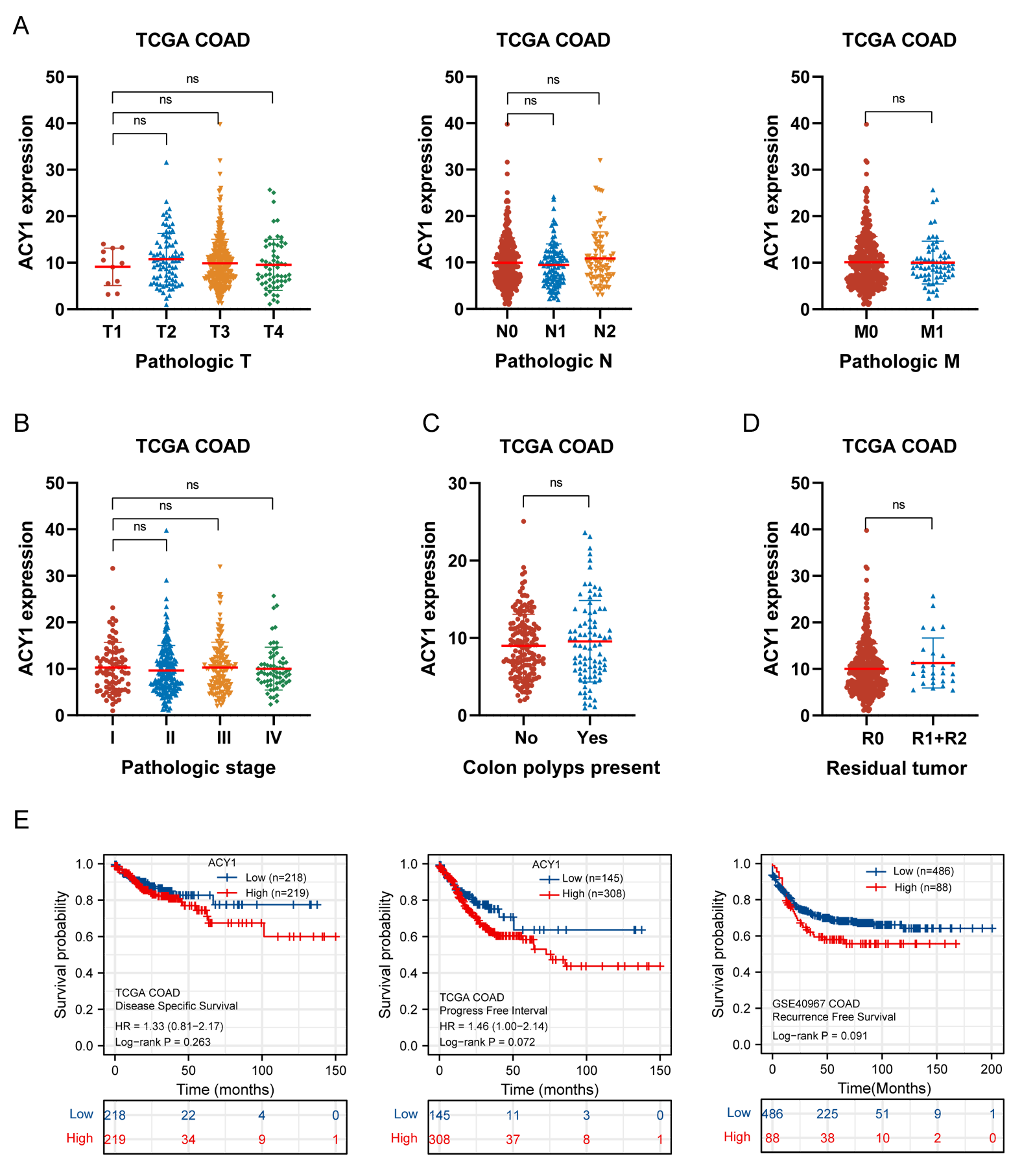

Supplement: Supplementary file 1 [file cancers-14-05704-s001.zip › Supplementary Figure S1.tif]

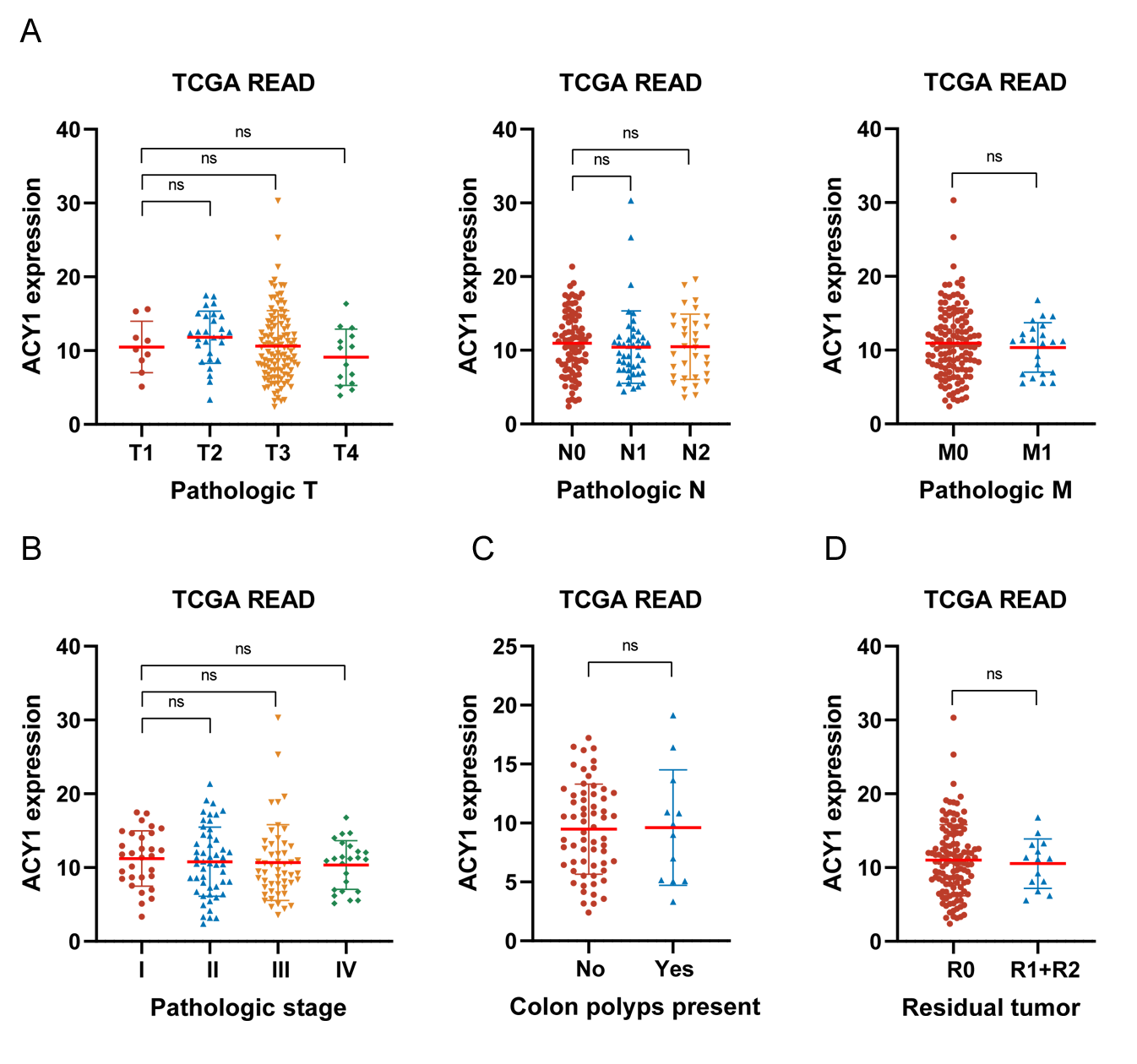

Supplement: Supplementary file 1 [file cancers-14-05704-s001.zip › Supplementary Figure S2.tif]

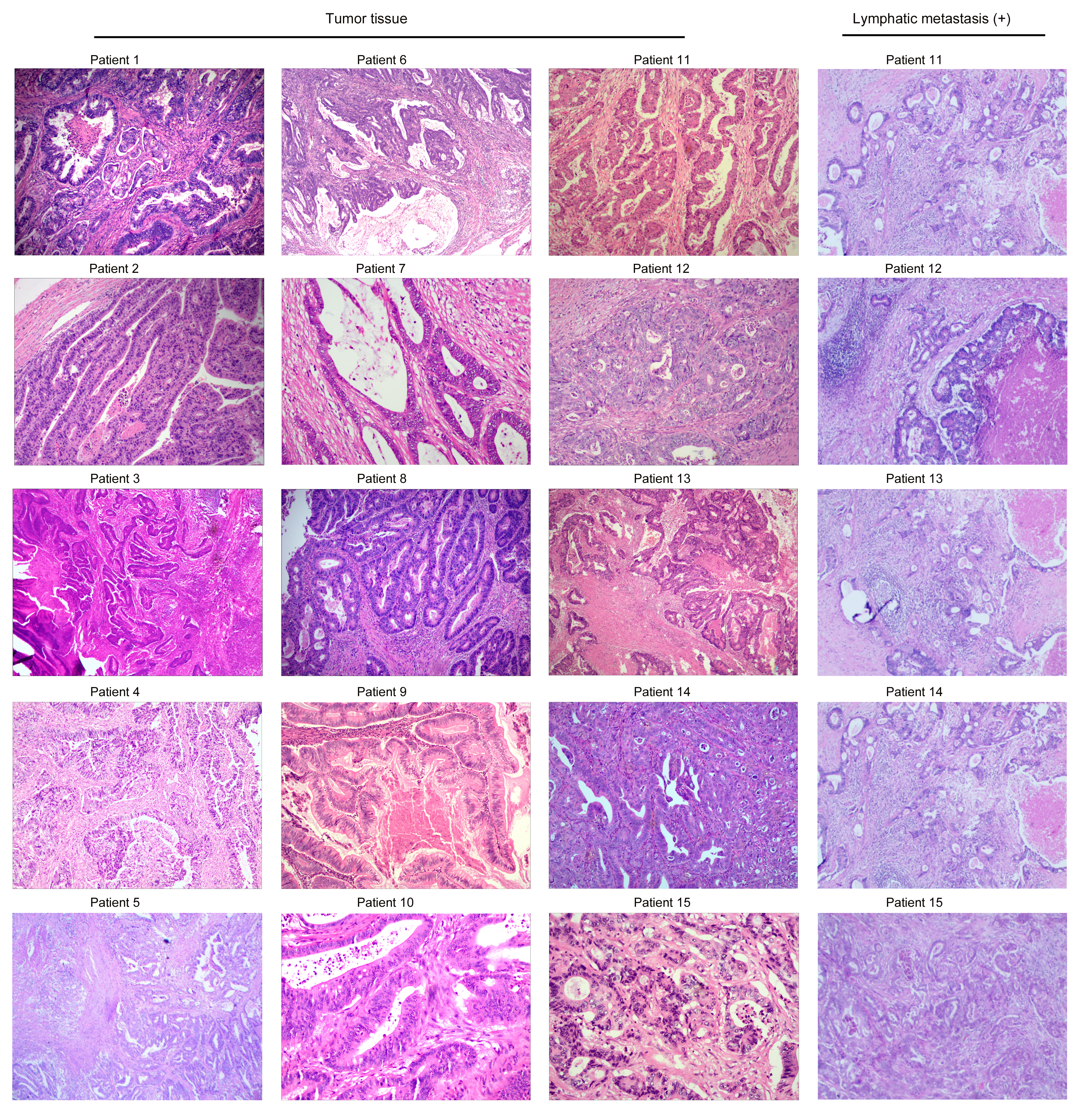

Supplement: Supplementary file 1 [file cancers-14-05704-s001.zip › Supplementary Figure S3.tif]
